# Enhancement of artificial magnetism via resonant bianisotropy

**DOI:** 10.1038/srep22546

**Published:** 2016-03-04

**Authors:** Dmitry Markovich, Kseniia Baryshnikova, Alexander Shalin, Anton Samusev, Alexander Krasnok, Pavel Belov, Pavel Ginzburg

**Affiliations:** 1 ITMO University, Nanophotonics and Metamaterials Department, St. Petersburg, 197101, Russia; 2 Tel Aviv University, School of Electrical Engineering, Tel Aviv, 69978, Israel

## Abstract

All-dielectric “magnetic light” nanophotonics based on high refractive index nanoparticles allows controlling magnetic component of light at nanoscale without having high dissipative losses. The artificial magnetic optical response of such nanoparticles originates from circular displacement currents excited inside those structures and strongly depends on geometry and dispersion of optical materials. Here an approach for enhancing of magnetic response via resonant bianisotropy effect is proposed and analyzed. The key mechanism of enhancement is based on electric-magnetic interaction between two electrically and magnetically resonant nanoparticles of all-dielectric dimer. It was shown that proper geometrical arrangement of the dimer in respect to the incident illumination direction allows flexible control over all vectorial components of the magnetic moment, tailoring the latter in the dynamical range of 100% and delivering enhancement up to 36% relative to performances of standalone spherical particles. The proposed approach provides pathways for designs of all-dielectric metamaterials and metasurfaces with strong magnetic responses.

Intrinsic magnetic polarizabilities of natural materials have strong frequency dependence with the fundamental cut-off in GHz range, originating from relatively low spin and orbital susceptibilities^1^. Recently, polarization currents in subwavelength structured loops, organized in ordered arrays, became sources of high-frequency artificial magnetism^2^,^3^. Nanostructured noble metals, supporting localized plasmon resonances could serve as building blocks for metamaterials with artificial magnetic polarizability^4^. However, inherent material losses set severe limitations on performances of such structures^5^. Another approach for obtaining magnetic optical response is to employ circular displacement currents in high-index dielectric nanoparticles^6^,^7^. This is the essence of so-called all-dielectric nanophotonics, which opened the way to control magnetic component of light at nanoscale without high dissipation, inherent for metallic nanostructures^8^,^9^,^10^,^11^,^12^,^13^,^14^. The “magnetic light” concept found use in various applications, such as nanoantennas^14^, quantum interface for NV-centers^16^, photonic topological insulators^17^, broadband perfect reflectors^18^, waveguides^19^, cloacking^20^, harmonics generation^14^, wave-front engineering, and dispersion control^21^, fluorescence enhancement^22^, and many more.

Magnetic response of a dielectric particle strongly depends on it’s refractive index, shape, and external environment. The eigen frequencies of electric and magnetic resonances could span the entire visible range. However, the magnitude of those multipole moments is limited by dispersion properties of optical materials.

Here an approach for tailoring magnetic response of dielectric nanoparticles via resonant bianisotropy is proposed. Microscopically, bianisotropy is the effect of magneto-electric coupling, where electric polarization induces magnetic and vice versa. The signature of this effect appears in the constitutive relations, e.g. the dependence of electrical induction also on magnetic field and magnetic induction on electric field^23^,^24^. Bianisotropy is used for achieving high values of effective polarizabilities in metamaterials^25^, unique properties of metasurfaces^24^,^26^, and designed directivity of nanoantennas^27^. Previously, it was shown that the interaction of dielectric nanoparticles with substrates may increase induced magnetic moment due to the effect of non-resonant bianisotropy^28^,^29^,^30^. Resonant interaction between a nanoparticle and a surface was analysed in ref. 31. The approach, proposed here, is based on electric-magnetic interaction between two resonant nanoparticles of a dimer (see Fig. 1). The nanoparticles were designed in the following way: the electric dipolar resonance of the bigger sphere spectrally overlaps with magnetic response of the smaller one. In this case, the effect of resonant bianisotropy is achieved: the resonant electric moment of the bigger nanoparticle induces the additional magnetic moment in the smaller one, tailoring its overall response. It is worth noting, that non-resonant overlapping regime was previously considered in ref. 32.

The manuscript is organized as follows: first, the optical properties of isolated spherical particles are briefly discussed in the context of the resonance tuning. Next, the analytical coupled dipoles formulation of the problem is developed and verified with numerical modeling. The expression describing the magnetic moment of the nanoparticle, with account for the bianisotropy, is derived. Finally, it is shown that proper geometrical arrangement of the dimer in respect to the incident illumination direction allows achieving additional vectorial component of magnetic polarization.

## Results and Discussions

### Coupled dipoles theory and numerical modelling

**Figure 2:**
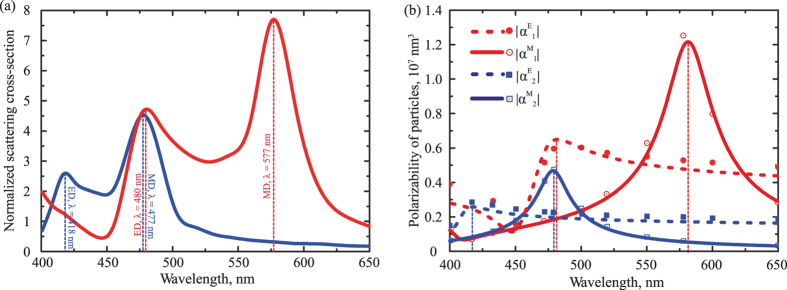
Optical properties of single silicon nanoparticles with radii *R*_1_ = 70 nm (red curves) and *R*_2_ = 52 nm (blue curves). (**a**) The scattering cross-section spectra, normalized to geometric cross-section (*πr*^2^). (**b**) Dispersion of particles polarizabilities. The curves correspond to analytical calculations, red circles and blue squares represent numerical results. Electric dipolar resonance of 70-nm radius particle overlaps with the magnetic dipolar resonance of 52-nm sphere at *λ* ≈ 480 nm.

In order to obtain the dimer design, properties of isolated components will be briefly discussed. First, isolated silicon sphere of radius *R*_2_ = 52 nm, having magnetic dipolar resonance at wavelength 477 nm, i.e. in the visible range is considered. The material dispersion of crystalline silicone (c-Si) is taken from ref. 33. The scattering cross-section of the nanoparticle has been calculated using FDFD (Finite-Difference Frequency Domain) simulations in CST Microwave Studio, and corresponding results as the function of wavelength are presented in Fig. 2(a) (blue curve). Electric dipolar resonance is blue shifted in respect to the magnetic one and appears in the spectrum at 418 nm wavelength. This Mie resonances hierarchy is red shifted when the radius of the nanoparticle is increased. It is worth noting, that high-order quadrupole resonances, also contributing to the scattering cross-section, are suppressed in this region due to perceptible losses of silicon. The bigger sphere with radius of *R*_1_ = 70 nm exhibits its electric resonance at the similar spectral position where 52-nm sphere exhibits resonant magnetic response (Fig. 2a, red curve). Resonance overlapping effect can be achieved in a single cylindrical or elliptical particle (e.g. ref. 34). The electric and magnetic polarizabilities which are associated with induced electric and magnetic moments have been also calculated (Fig. 2b). Results of numerical simulations (dots) are in good agreement with analytical Mie theory (curves) verifying the validity of the numerical tool. Values of those electric and magnetic moments will be subsequently used in the analytical model, based on discrete dipole approximation.

Next, the scattering of a plane wave on all-dielectric dimer will be analyzed. The electromagnetic scattering problem could be solved by employing Coupled Electric and Magnetic Dipole Approximation^29^,^35^ (CEMDA). In this method, complex nanostructures are represented by converging series of point electric and magnetic dipoles, while the problem of two spheres could be approximated by only two. This approach is particularly accurate, if the gap between the spheres is bigger than their radii^3^, however it could be still applicable for the case of small separation distances, as it was shown in ref. 29. The goal of the subsequent analytical analysis is obtaining a simple formulation, underlining the interference phenomena, affecting the magnetic dipole moment of the smaller particle and revealing the bianisotropic nature of the interaction. The impact of higher order modes has been taken into account using the full-wave numerical calculations. Following the CEMDA method, total electromagnetic fields [both electric (**E**) and magnetic (**H**)] were decomposed into incident and scattered components:


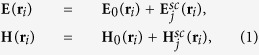


where indices *i*, *j* = 1, 2 (*i* ≠ *j*) denote the first (bigger) and the second (smaller) nanoparticle, respectively, **E**_0_ and **H**_0_ are electric and magnetic fields of the incident plane wave, **E** and **H** are the total electric and magnetic fields, 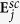 and 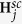 are the electric and magnetic fields scattered by the nanoparticle with index “*j*”, **r**_*i*_ is a radius-vector of “*i*”-nanoparticle’s center. Both particles are polarized by the incident field, as well as by the scattered one:


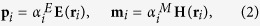


where 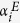 and 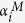 are the electric and magnetic polarizabilities of a single particle “*i*”:


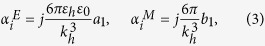


and *ε*_*h*_ is the dielectric constant of surrounding medium, *ε*_0_ is the vacuum permittivity, *j* stands for the imaginary unit. The coefficients *a*_1_ and *b*_1_ are the first order Mie scattering coefficients, and are given by: *a*_1_(*λ*) = [*A* − *B*]/[*C* − *D*], *b*_1_(*λ*) = [*Bn*^−2^ − *A*]/[*Dn*^−2^ − *C*], where the following notation is introduced:


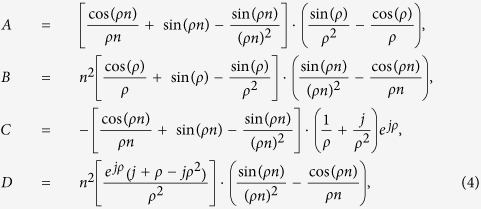


where 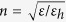, *ρ* = *k*_*h*_*R*_*i*_, *R*_*i*_ - radius of the sphere “*I*”, *ε* - the permittivity of the material (silicon, here), 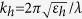 is the wave number in the surrounding medium, and *λ* is a free-space wavelength.

The values of single particle polarizabilities, calculated using Eqs. (3) and (4), are in a perfect agreement with our numerical calculations [see Fig. 2(b)]. Thus, the scattered fields of the dipoles can be obtained through the Green’s function of a point dipole in a free-space 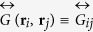
35, following the CEMDA^3^:


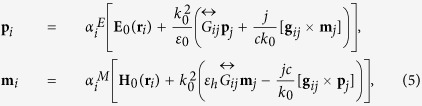


where *k*_0_ and *c* are the wavenumber and speed of light in vacuum. The Green’s function of a point dipole in a free-space is given by:


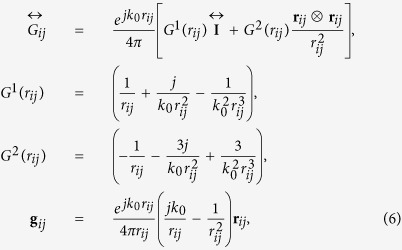


where **r**_*ij*_ = **r**_*i*_ − **r**_*j*_ is the radius-vector connecting the first dipole (coordinates origin, or the center of the bigger particle) with the second one (center of the smaller particle), 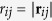. The tensor 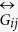 and the vector **g**_*ij*_ have the following symmetry of indices permutation: 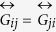 and **g**_*ij*_ = −**g**_*ji*_.

**Figure 3:**
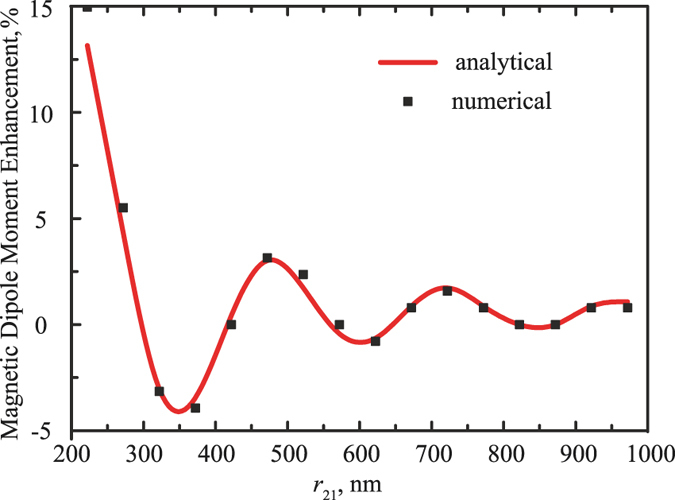
Enhancement of magnetic dipole moment of the smaller nanoparticle for the case *θ* = *π*/2, *φ* = 0. The red curve corresponds to the CEMDA solution, while black squares are the results of numerical full-wave simulation.

Both nanoparticles, being isolated, have 3-fold degenerated (magnetic and electric) dipolar resonances, oriented along the unit vectors of a Cartesian coordinate system. The excitation of an isolated sphere is solely defined by the polarization of the incident wave – for example, linearly polarized beam will excite only one of the 3 components of the dipolar mode. However, the geometry of the coupled dipoles together with the excitation (not necessarily coinciding with one of the symmetry axis of the system) will break the degeneracy and, as the result, all three vectorial components should be taken into account. The resulting set of equations can be solved analytically by means of the matrix inversion or numerically in the same fashion. In order to verify the validity of the proposed theoretical model, a particular case, where the system of Eqs. (5) has a simple and intuitive solution, was considered. Hereinafter the angles are introduced in accordance with a standard definition of the spherical coordinate system: *θ* is the angle between the vector and the axis *z, φ* is the angle between the vector projection on the *x* − *y* plane and *x* axis. Arranging the nanoparticles along the x-axis and exciting the system with plane wave linearly polarized along y-axis with angles being *θ* = *π*/2 and *φ* = 0, the set of 12 coupled equations was reduced just for 4, since the symmetry considerations allow the moment components to be induced only along z-axis (magnetic dipole) and y-axis (electric dipole). This set of coupled equations has particularly simple solution, which agrees well with the numerical calculations. The values of the magnetic moment enhancement, calculated both analytically and numerically as the function of the distance *r*_12_ between nanoparticles are shown in Fig. 3. It is clearly seen, that the analytical and numerical models agree well with each other, 15% enhancement of the magnetic moment of the single nanoparticle, as the result of the bianisotropic coupling, can be also observed. For clear understanding of the oscillatory behaviour of the magnetic moments, the simplest model, when the polarizabilities 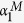 and 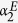 are supposed to be zero, was considered. In this case the simple formula can be obtained, underlining the bianisotropic nature of the effect:


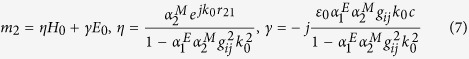


The qualitative analysis of magnetic moment *m*_2_ enables observing the clear interference phenomena–the direct excitation of magnetic dipole by the plane wave 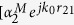 term in Eq. (7)] and the contribution of the scattered field through the term 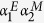. Thus, the oscillation behavior of magnetic moment enhancement is the result of the interference phenomenon (see Fig. 3). For larger distances the coupling between the particles becomes weaker and converges to the value of the isolated particle. An almost perfect fit of full numerical modeling with the CEMDA method enables to use the latter for analysis of more complex structures without involving full wave simulations. This approach will be subsequently employed.

### Vectorial structure of magnetic moments

**Figure 4:**
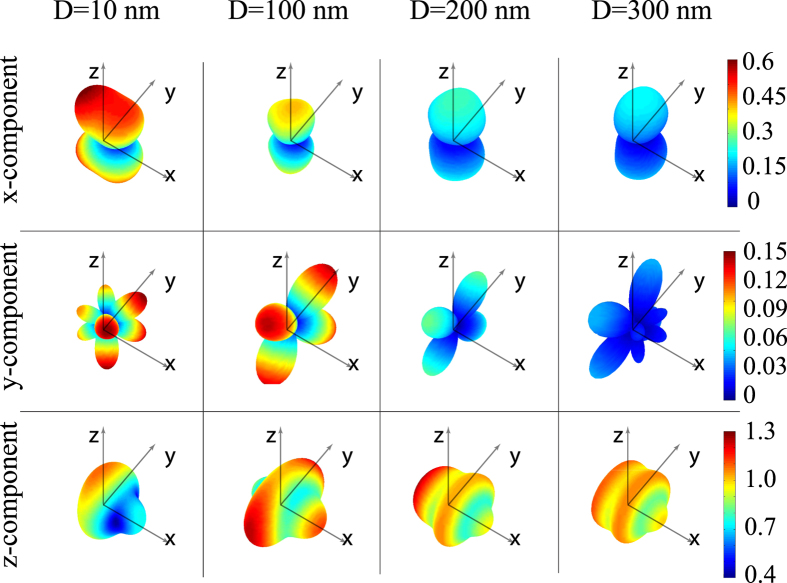
The three-dimensional angular dependencies of the relative increase of the smaller nanoparticle’s magnetic moment 
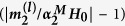
 for all vectorial components *l* = *x*, *y*, *z* for the different distances *D*  = 10, 100, 200, and 300 nm.

**Figure 5:**
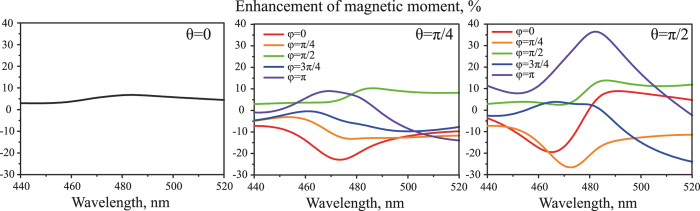
The spectral dependencies of the enhancement of magnetic dipole moment
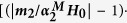

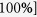
 of the smaller nanoparticle for *D* = 168 nm and different angles (*φ*, *θ*). The data for *θ* ∈ (*π*/2, *π*) and *φ* ∈ (*π*, 2*π*) repeat the presented results, therefore they are omitted. For the case of *θ* = 0 the lines for all *φ* coincide.

In the subsequent studies the illumination was chosen to propagate along *x*-axis and being polarized along *y*. There are three geometrical parameters, affecting the magnetic moment of the smaller particle: the distance between the spheres’ centres *r*_12_ = *R*_1_ + *R*_2_ + *D*, and the angles *θ* and *φ*. The angular dependence of the induced magnetic moment components of the smaller particle for 3 characteristic separation distances *D* = 10, 100, 200, and 300 nm (gap between the nanoparticles’ surfaces) will be studied next. In the case of noninteracting nanoparticles there is the only one non-zero component of nanoparticle’s magnetic moment 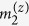
–codirected with the magnetic field. In the case of bianisotropic coupling there are also *x*- and *y*- non-zero components of the magnetic moment 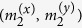. Figure 4 shows the three-dimensional angular dependencies of normalized magnetic moment components relative increase (
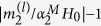
, *l* = *x*, *y*, *z*) of the smaller nanoparticle, where *α*_2_^*M*^ is the polarizability of the single smaller nanoparticle. These results have been obtained by exact solving of Eqs. (5), taking into account the electric and magnetic responses of both nanoparticles. Both induced magnetic and electric moments of the smaller nanoparticle were calculated using the theoretical model CEMDA. It could be seen, that in all the cases the induced components of the magnetic dipole are smaller than the main one. Those additional vectorial components of magnetic moments increase when the distance between the particles goes down and may grow up by 50% for *D* < 100 nm. The model validity for 10 nm separating distance between the particles was verified by the numerical simulations. When the distance between the particles increases, secondary vectorial components of magnetic moment goes down. Therefore, the overall variation of magnetic moments is mainly determined by its *z*-component for large separation distances. Diagrams of *z*-component of the magnetic moment are asymmetrical and their forms are dissimilar for different separation distances between the particles (*D*). For *D* ≥ 300 nm enhancement of magnetic moment is weak, because of the vanishing coupling between the particles. For *D* > 2 *μ*m this diagram is symmetrical with a good accuracy, as it replicates the performance of the isolated particle. For distances *D* = 10, 100 nm the maximum of magnetic moment corresponds to the cases (*θ* = *π*/2, *φ* = *π*/2 and *φ* = 3*π*/2), and for *D* = 200, 300 nm maximum of magnetic moment corresponds to the case (*θ* = *π*/2, *φ* = *π*). It should be noted that the case (*θ* = *π*/2, *φ* = *π*/2 and *φ* = 3*π*/2) corresponds to magnetic dipoles coupling only, while the case (*θ* = *π*/2, *φ* = *π*) corresponds to electric-magnetic dipoles coupling only. Additional analysis shows that the maximum of relative magnetic moment enhancement is nearly 36% and it is achieved for *D* = 168 nm for the case of (*θ* = *π*/2, *φ* = *π*). The performance of the dimer with this separation distance will be investigated in details hereafter. Enhancement of the magnetic moment of smaller nanoparticle 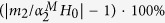 for different angular arrangements (the distance *D* = 168 nm is kept constant) is shown in the Fig. 5. These dependences show the resonance character of the effect. Moreover, the maximum value is achieved at the wavelength of 480 nm i.e. at the electric dipole resonance of bigger nanoparticle and magnetic dipole resonance of smaller one underlining the impact of the resonant nature.

### Outlook and conclusions

Coupled particles approach for controlling magnetic moments of nanoscale spheres was proposed. While standalone nanoparticles allow obtaining dipolar and high-multipolar resonant responses at the desired wavelength in the visible range by specifying their radii and materials, a system of two coupled nanoparticles possesses more degrees of freedom. Altering the radii of both nanoparticles allows investigating the impact of all the combinations of multipolar coupling effects on the properties of the system. Naturally, the amplitude of the effects is dependent on their mutual displacement, and it decreases at larger distances between the particles, as it was proved both analytically and numerically in the case of dipolar magnetic-electric coupling. Symmetry considerations allow exciting only one dominant induced electric and magnetic moment component when a plane wave is incident upon a single nanoparticle. It was shown, that for the nanoparticle system, altering the spherical angles and distance between nanoparticles allows to excite all vectorial components of magnetic moment simultaneously. The secondary components being up to 50% of the amplitude value of the dominant one. Furthermore, these parameters also define the spectral position of dips and peaks of the dominant electric moment components and amplitude values, allowing full on-demand control of the electromagnetic properties of the system of coupled nanoparticles. The proposed approach can find use in designs of more complex structures such as all-dielectric metamaterials and metasurfaces with strong magnetic responses.

## Additional Information

**How to cite this article**: Markovich, D. *et al.* Enhancement of artificial magnetism via resonant bianisotropy. *Sci. Rep.* **6**, 22546; doi: 10.1038/srep22546 (2016).

## Figures and Tables

**Figure 1:**
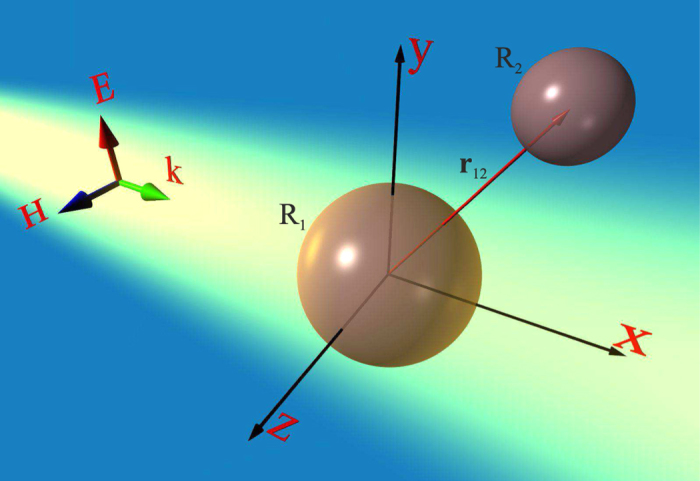
The geometry of the bianisotropic all-dielectric dimer. The system consists of two dielectric nanoparticles separated by a distance **r**_12_. The sizes of nanoparticles are *R*_1_ (bigger nanoparticle) and *R*_2_ (smaller nanoparticle). Electric dipole resonance of the big sphere overlaps with magnetic dipole resonance of the small one. The dimer is excited by a *y*-polarized plane wave propagating along *x*-axis.
