# Corrigendum: Enhancement of artificial magnetism via resonant bianisotropy

**DOI:** 10.1038/srep24003

**Published:** 2016-04-20

**Authors:** Dmitry Markovich, Kseniia Baryshnikova, Alexander Shalin, Anton Samusev, Alexander Krasnok, Pavel Belov, Pavel Ginzburg

Scientific Reports 6: Article number: 2254610.1038/srep22546; published online: 03042016; updated: 04202016.

The Acknowledgements section in this Article is incomplete.

“This work has been supported by the Russian Fund for Basic Research within the project 16-52-00112. The calculations of multipole moments and fields has been supported by the Russian Science Foundation Grant No. 16-12-10287.”

should read:

“This work has been supported by the Russian Fund for Basic Research within the project 16-52-00112. A.S.S. acknowledges the support of the President of Russian Federation in the frame of Scholarship SP-4248.2016.1. The calculations of multipole moments and fields has been supported by the Russian Science Foundation Grant No. 16-12-10287.”

